# An integrated, systems-wide approach is needed for public–private partnerships to drive genetic innovation in crops

**DOI:** 10.1371/journal.pbio.3002181

**Published:** 2023-07-06

**Authors:** Catherine Feuillet, Kellye Eversole

**Affiliations:** 1 INARI Agriculture, Cambridge, Massachusetts, United States of America; 2 Eversole Associates, Arlington, Massachusetts, United States of America; University of California, Davis, UNITED STATES

## Abstract

To meet the agricultural challenges caused by climate change and a growing population, we need to improve crop production. This Perspective calls for more and better public–private partnerships to accelerate discoveries in crop research.

## Teaser

Public–private partnerships are key to successfully translate knowledge to products, but current frameworks do not foster the systems-wide approach required to improve crops to meet the agricultural production challenges of the 21st century.

In “Sapiens: a brief history of Mankind” [1], Yuval Noah Harari highlights that “Homo sapiens have developed an unprecedented and unique ability among animals to cooperate flexibly in large numbers and imagine things collectively.” What a great way to describe research! The history of science is filled with examples of collaborations that solved societal problems and advanced humanity. As we are challenged today in our ability to keep our humanity in the face of our own growth and impact on the planet, we urgently need to activate and foster our collaborative skills for the greater good.

Scientific advancements and, more specifically, innovation in plant genetics have played a key role over the past 100 years in supporting crop improvement, culminating with the green revolution and advances in plant biotechnology that contributed to significant yield increases in major crops [2]. While very successful in increasing overall food production, this came with high environmental costs that we can no longer afford. The dilemma we face today of how to increase agricultural productivity while reducing agriculture’s environmental footprint under the pressure of a rapidly changing climate is one of our most complex societal and technological challenges. The urgency of the challenge requires a significantly faster pace of innovation in crop genetics to support the sustainable development of products that perform optimally in these changing environments with minimal use of natural resources and inputs. Further, these products will need to reach the market in 2 to 3 years rather than 10 to 15 years, empower a diverse ecosystem that provides farmers with choices, and enable all actors of the production system to capture value for their labour and investments.

Innovation in crop genetics is struggling to meet these urgent needs, primarily because of the difficulty in holistically addressing the complexity of the biomes in which the crops grow (i.e., the phytobiomes) and in adroitly managing the innovation ecosystems. For example, for more than 50 years, plant research has relied heavily on model plants [3] and experiments in controlled environments, which inadequately represent the full complexity of the biomes, thereby creating an innovation gap that has been very difficult to overcome. Furthermore, innovation has often been achieved by advancing research projects in isolation or in parallel within academia and industry. The inherent difficulty in managing and reconciling the differences in goals, incentives, processes, and ways of working between academia and industry, as well as the reluctance to address upfront points of contention such as intellectual property and data sharing, often narrows the scope of joint projects, thereby limiting the ambition of these partnerships.

The urgent need to address agricultural production challenges calls for a renewed way of working, specifically, for a more collective and integrative manner with efficient public–private partnerships (PPP) at its heart. The pharmaceutical industry faced similar challenges in the early part of this century [4]. To increase innovation, reduce the translational gap (from fundamental research to products), and accelerate the time needed to deliver solutions to patients, mixed models of research and development partnerships were established between industry and academia. This resulted in large, well-funded, and impactful initiatives that injected innovation into the system. How can these experiences inspire and guide us to improve the effectiveness of PPPs in crop improvement? Beyond the need for a strong common sense of purpose and the willingness to acknowledge and actively align each other’s core objectives, best management practices (such as an appropriate consortium management structure), and an independent, neutral, and empowered facilitator acting as a platform to manage calls for projects and government funding are cited as key success factors [5,6].

We believe that the time is ripe for the agricultural research world at large to address the looming productivity gap [7] through a systems-wide approach with the same urgency, ambition, and scale as that adopted by the biomedical world. We also need to address the additional imperative of improving multiple crops concurrently without diluting already scarce resources, especially those in the public funding systems [8]. Systems approaches that rely on problem solving by interdisciplinary or, even better, transdisciplinary teams and that are managed through organizational integration are especially relevant to the crop improvement ecosystem, given the diversity of players and of interactions between and for each crop. While there is a history of PPPs in support of crop improvement and some success stories [8], they have very rarely traversed disciplinary boundaries or managed the integration dimension. They also suffered from myopic visions and a lack of long-term and large-scale funding that resulted in an estimated rate of 99% of new agri-food technologies not reaching markets [8].

It is time for a bold, broad, integrated systemic approach in PPPs for crop improvement that will establish shared grand visions for each crop. We need strategies that integrate the “product-driven” framework deployed in industry to aggressively tackle the current bottlenecks from ideation to commercialization (Table 1), focus basic and applied research funding on crops as the new model plants to reduce the time to commercialization, and enhance the connections and alignment between the public and private partners of the ecosystem through professional and dedicated neutral coordinators that can manage diverse sources of funding. Private industry needs to be incentivized to share their data so that novel machine learning capabilities can be deployed to boost knowledge building in crop biology and increase overall competitiveness, as has been done in the Melloddy project and elsewhere in biomedicine [9]. The contributions of all public and private partners need to be rewarded with a return on investments by incorporating mechanisms to share intellectual property and revenues to ensure long-term funding and ambitious goals of the PPP. We must embrace transdisciplinary, translational science that breaks down silos between disciplines and the different levels of organization within the plant biological system [10]. Finally, we need to integrate within these partnerships highly exploratory activities to fuel innovation [11] and work experience in industry and government as key components of educational curricula that will enable swift career shifts and better prepare students for success in translational science.

**Table 1:** Product-driven approach to ensure alignment and efficiency in public–private partnerships (PPPs) for crop improvement.

	Define ideotypes	Create knowledge and tools	Integrate knowledge and tools into products	Provide to users
**Challenges/Bottlenecks**	• Define ideotypes in a way that guides the creation of knowledge (which performance for which environment/geographies in which genetics etc.) • Access diversity and elite material • Access existing performance data	• Very limited understanding of crop biology and complex traits that underpin Performance x Environment • Linear, non-systemic, siloed approaches • Intellectual Property (IP), Freedom To Operate • Data sharing • Translation from model to crops • Translation from controlled environment to fields • Translation from *in cellulo* assays to *in planta*	• Plant transformation efficiency and feasibility in elite material • Access and cost of field trials at scale • Reproducibility and scalability of technologies	• Regulatory framework • License to operate, public acceptance
**Partners’ role**	• Private: support definition of ideotypes of interest, share data • Public: provide access to diversity panels	• Private: provide in-kind or fund activities, enable publications and release of data in shared databases, ensure IP is enabling and not stifling innovation, provide access to large-scale technology platforms • Public: ensure rewarding systems integrate success criteria beyond bibliometric indexes, prioritize fundamental research in crops, foster translational science education that includes industry experience, explore new technologies through transdisciplinary approaches, and train students in scientific creativity	• Private: use knowledge and tools in own product pipelines • Public: use knowledge and tools in own research and breeding programs	• Private: ensure that product reaches market and generates revenues, explain value of PPP to customers • Public: provide educational programs for public (e.g., genetic literacy at Cornell)
**PPP activation mechanism**	• Fund workshops and meetings to establish the grand vision for the project • Condition the receipt of project funding upon the co-creation of the ideotypes	• Design funding mechanisms that reward systems and transdisciplinary approaches • Define technology access and transfer • Integrate IP and publication timeline management within the PPP agreement at the onset	• Manage and facilitate access to advanced technologies within PPPs • Enable data sharing to feed back into the creation of knowledge, tools, and activities	• Join forces to work with regulatory agencies • Establish mechanisms to generate royalties on product sales to maintain funding of the PPP • Join forces in communicating about the PPP achievements

While daunting, we see some signs for hope. With the increasing recognition by industry of the benefits of PPPs to fuel innovation, several industry-led efforts have contributed to improve important crops by sharing knowledge, resources, and products. Recently, driven by the promises of genome editing for transforming crop improvement and crop health, a number of startups have emerged. They are very often led by people with a mixed background in academia and industry and a deep understanding of the value of collaboration between the 2 worlds. Further, initiatives like Crops Of The Future, supported by the Foundation for Food and Agricultural Research, are heading in the right direction. Here, decisions to tackle bottlenecks with investments in key crops are taken collectively and are supported by an independent facilitator in a framework established at the onset to address friction points between parties. While promising, these efforts are not yet at the scale that will result in the step-change needed for crop production. Thus, it is urgent that the crop improvement community at large embraces and further multiplies PPPs to tackle together one of the biggest challenges of our times.

## References

[1] Harari YN. Sapiens: A Brief History of Humankind. New York; Harper, 2015.

[2] Qaim M. Role of New Plant Breeding Technologies for Food Security and Sustainable Agricultural Development. Appl Econ Perspect Policy. 2020;42(2):129–150. doi: 10.1002/aepp.13044

[3] Provart NJ, Alonso J, Assmann SM, Bergmann D, Brady SM, Brkljacic J, et al. 50 years of Arabidopsis research: highlights and future directions. New Phytol. 2016;209(3):921–944. doi: 10.1111/nph.13687 26465351

[4] Tralau-Stewart CJ, Wyatt CA, Kleyn DE, Ayad A. Drug discovery: new models for industry–academic partnerships. Drug Discov Today. 2009;14(1–2):95–101. doi: 10.1016/j.drudis.2008.10.003 18992364

[5] Said M, Zerhouni E. The role of public-private partnerships in addressing the biomedical innovation challenge. Nat Rev Drug Discov. 2014;13(11):789–790. doi: 10.1038/nrd4438 25359362

[6] Pronk JT, Lee SY, Lievense J, Pierce J, Palsson B, Uhlen M, et al. How to set up collaborations between academia and industrial biotech companies. Nat Biotechnol. 2015;33(3):237–240. doi: 10.1038/nbt.3171 25748909

[7] Xu Y, Li P, Zou C, Lu Y, Xie C, Zhang X, et al. Enhancing genetic gain in the era of molecular breeding. J Exp Bot. 2017;68(11):2641–2666. doi: 10.1093/jxb/erx135 28830098

[8] Smyth SJ, Webb SR, Phillips PWB. The role of public-private partnerships in improving global food security. Glob Food Sec. 2021;31:100588. doi: 10.1016/j.gfs.2021.100588

[9] Data sharing in the age of deep learning. Nat Biotechnol. 2023;41(4):433. doi: 10.1038/s41587-023-01770-3 37020134

[10] Passioura JB. Translational research in agriculture. Can we do it better? Crop and Pasture Science. 2020;71(6):517–528. doi: 10.1071/CP20066

[11] Yanai I, Lercher MJ. Make science disruptive again. Nat Biotechnol. 2023;41(4):450–451. doi: 10.1038/s41587-023-01736-5 36973558

